# Immunogenicity and Reactogenicity of the Booster Dose of COVID-19 Vaccines and Related Factors: A Panel Study from the General Population in Serbia

**DOI:** 10.3390/vaccines10060838

**Published:** 2022-05-25

**Authors:** Maja Stosic, Marija Milic, Milos Markovic, Ivana Kelic, Zoran Bukumiric, Marko Veljkovic, Darija Kisic Tepavcevic, Vladan Saponjic, Dragana Plavsa, Sofija Jovanovic, Verica Jovanovic

**Affiliations:** 1 Institute of Public Health of Serbia “Dr Milan Jovanovic Batut”, Belgrade, Dr Subotica 5, 11000 Belgrade, Serbia; marija_milic@batut.org.rs (M.M.); ivana_kelic@batut.org.rs (I.K.); marko_veljkovic@batut.org.rs (M.V.); vladan_saponjic@batut.org.rs (V.S.); dragana_djordjevic@batut.org.rs (D.P.); 2 Department of Epidemiology, Faculty of Medicine, University of Pristina Temporarily Seated in Kosovska Mitrovica, 38220 Kosovska Mitrovica, Serbia; 3 Faculty of Medicine, Institute for Microbiology and Immunology, University of Belgrade, Dr Subotica 1, 11000 Belgrade, Serbia; milos.markovic@med.bg.ac.rs; 4 Faculty of Medicine, Institute for Medical Statistics and Informatics, University of Belgrade, Dr Subotica 9, 11000 Belgrade, Serbia; zoran.bukumiric@med.bg.ac.rs; 5 Faculty of Medicine, Institute of Epidemiology, University of Belgrade, Visegradska 26A, 11000 Belgrade, Serbia; darijakt@gmail.com; 6 Faculty of Medicine, University of Belgrade, Dr Subotica 9, 11000 Belgrade, Serbia; jovanovicdsofija@gmail.com

**Keywords:** COVID-19, Serbia, vaccination, booster dose, mix and match strategies, immunogenicity, reactogenicity

## Abstract

The Republic of Serbia applied the booster dose of the following COVID-19 vaccines: BNT162b2 mRNA (Pfizer-BioNTech), Sinopharm BBIBP-CorV (Vero Cell^®^), Gam-COVID-Vac (Sputnik V) and ChAdOk1 nCoV-19 (AstraZeneca). We aimed to examine the immunogenicity and reactogenicity of the booster dose and identify factors related to immune response and adverse events. Panel study, conducted during August and September 2021, included 300 persons receiving the booster dose at the Institute of Public Health of Serbia. Blood samples were taken on the day of receiving the booster dose, and after 7 and 28 days. When applying homologous regimen, the average increase in anti-spike immunoglobulin G was 8782.2 (after 7 days), 1213.9 after 28 days, while 9179.5 (after 7 days) and 16,728.1 after 28 days of heterologous regimen. Sinopharm BBIBP-CorV (*p* < 0.001) and Sputnik V (*p* < 0.001), age 65 and over (*p* = 0.001) and currently smoking (*p* < 0.001) were independently associated with lower levels of anti-spike immunoglobulin G. Female sex (OR = 1.77; 95%CI = 1.01–3.12), previous COVID-19 infection (OR = 3.62; 95%CI = 1.13–11.63) and adverse events after the second dose (OR = 2.66; 95%CI = 1.33–5.32) were independently associated with intense systemic adverse events 7 days after. Booster dose significantly increased antibodies titers, especially 28 days after heterologous regimen, without a significant increase in reactogenicity.

## 1. Introduction

The implementation of COVID-19 vaccines has become the main weapon in the battle of slowing down the coronavirus disease pandemic and reducing morbidity and mortality worldwide [1,2]. Israel was the first country to record a significant decrease in COVID-19 cases in early 2021 due to the mass immunization, but also the first country to question the immunization achievements due to breakthrough infections among fully vaccinated people [3,4,5]. Two main reasons for breakthrough infection occurrence are the emergence of new SARS-CoV-2 variants that escape immunity and waning immunity of the vaccines over time [1,6,7,8].

Policymakers of many countries decided to administer a booster dose of COVID-19 vaccines and apply mix and match strategies based on the following reasons: lower efficacy of certain vaccines, waning immunity and the increase in new COVID-19 cases from June 2021 [1,9,10,11,12,13]. The Republic of Serbia was among the first countries to start applying the booster dose of COVID-19 vaccines, allowing fully vaccinated individuals to receive any of the four COVID-19 vaccines available in the country: BNT162b2 mRNA (Pfizer-BioNTech), Sinopharm BBIBP-CorV (Vero Cell^®^), Gam-COVID-Vac (Sputnik V) and ChAdOk1 nCoV-19 (AstraZeneca), six months after receiving the second dose (mix and match strategies) [14].

The results of the first studies on the immunogenicity and reactogenicity of the booster dose are encouraging, showing high immunogenicity of the homologous or heterologous application regimen of all tested vaccines, with good tolerability [15,16,17,18]. Additionally, greater effectiveness and safety profile of the COVID-19 vaccine is associated with a greater acceptance of vaccination, especially among men, younger age groups, those with high vaccine hesitance and with lower incomes [19,20,21].

A recent study from Israel showed that the effectiveness of the booster dose of the BNT162b2 mRNA (Pfizer-BioNTech) vaccine after 7 days was 93% for hospital admission, 92% for severe illness and 81% for death from COVID-19 compared to receiving only two doses of the same vaccine 5 months ago [22]. Most of the current studies examined the immune response, reactogenicity and effectiveness of BNT162b2 mRNA (Pfizer-BioNTech), mRNA-1273 (Moderna) or ChAdOx1 nCoV-19 (AstraZeneca) given as a booster dose within a homologous or heterologous regimens [16,17,18]. However, there are only a few studies comparing the safety and immunogenicity of the combined application of vaccines approved by the European Medicines Agency with BBIBP-CorV (Sinopharm) or Gam-COVID-Vac (Sputnik V) vaccines [15,18]. Therefore, the aim of this study was to examine immunogenicity and reactogenicity after the application of different COVID-19 vaccines as a booster dose within the mix and match strategies, as well as to identify factors related to immune response and adverse events following vaccination.

## 2. Materials and Methods

### 2.1. Study Design

We performed a panel study from 23 August to 20 September 2021 at the Institute of Public Health of Serbia “Dr Milan Jovanovic Batut”. The study was composed of a prospective cohort and three cross-sectional studies. The first cross-sectional study was conducted at the beginning of the study (before receiving a booster dose), the second was conducted seven days later, while the third cross-sectional study was performed on the 28th day after receiving a booster dose of the selected vaccine against COVID-19.

### 2.2. Sample and Procedure

The sample size was calculated using the program G-power 3.1.6 [23] to detect the effect size of 0.33 in the analysis of variance of repeated measurements. Assuming patient loss during the study duration, or exit from the study of 20%, the final minimum sample size was 159 subjects. The magnitude of the effect was obtained based on the assumed ratios of the values of the explained and residual variance from 0.1 to 0.9. All individuals who applied for a booster dose vaccination at the Institute of Public Health of Serbia “Dr Milan Jovanovic Batut” were consecutively included in the study until the sample size was achieved. The study included 300 consecutive respondents who agreed to participate in the study. The inclusion lasted for 5 days. Only 7 respondents (respond rate 97.7%) refused to participate in the study. There were no vaccination appointments. Coming to the vaccination was voluntary and accidental. We avoided selection bias by the enrollment of consecutive respondents.

Individuals with mental inability to understand the goals and study procedure were excluded as well as the ones having temporary or permanent contraindication for vaccination determined by a medical doctor in accordance with the National Methodological Guidelines for COVID-19 vaccination [24].

Citizens of the Republic of Serbia had the possibility to choose four vaccines offered for primary vaccination: BNT162b2 mRNA (Pfizer-BioNTech), Sinopharm BBIBP-CorV (Vero Cell^®^), Gam-COVID-Vac (Sputnik V) and ChAdOk1 nCoV-19 (AstraZeneca). All vaccines were administered in a homologous two-dose regimen with an interval of three weeks between two doses except for ChAdOk1 nCoV-19 when the interval between two doses was 12 weeks. From August 2021, all citizens of the Republic of Serbia over the age of 18 could receive the booster dose. The minimum interval from the second dose of the primary vaccination series to the booster dose was 6 months. In addition, all citizens could decide to receive any of the four available vaccines in the Republic of Serbia as a booster dose, regardless of the type of vaccine they had received during the primary vaccination. Depending on whether the participants of our study chose the same or a different type of booster dose of vaccine in relation to the primary vaccination, they were classified in the group with homologous regimens of vaccine administration, or in the group with heterologous regimens.

### 2.3. Measurements

We conducted an in-person survey facilitated by health workers who were trained on survey administration prior to study initiation. The collection of epidemiological data was carried out at the beginning of the study (before booster dose administration) using a structured questionnaire consisting of closed-ended questions, covering the following topics: socio-demographic characteristics of the respondents, habits and behaviors, concomitant diseases and previous COVID-19, immunosuppressive treatment, allergy to food and drugs, the type of vaccine received as the primary series of COVID-19 vaccination and adverse events after the primary series of COVID-19 vaccination.

### 2.4. Sample Collection and Measurement of Immunogenicity

Blood samples were collected from the participants in order to determine titers of anti-spike immunoglobulin G (anti-S-IgG) measured by immunoassay on three occasions (before booster doses, 7 and 28 days after the booster dose). Sample analysis was performed in the microbiology laboratory of the Institute of Public Health of Serbia. Antigen-specific humoral immune response was analyzed using Abbott Architect SARS-CoV-2 IgG Chemiluminescent microparticle immunoassay (CMIA), (Abbott Diagnostics, Chicago, IL, USA), performed on theAbbott Architect i2000SR platform. The IgG value was expressed in arbitrary units per mL of serum (AU/mL). According to the manufacturer’s instructions, IgG values equal and above 50 AU/mL were considered positive.

### 2.5. Reactogenity of Booster Dose

Reactogenicity data were collected on days 7 and 28 after the COVID-19 booster vaccination by interviewing the participants using the questionnaire related to local and systemic adverse events. Local adverse events were pain, redness, swelling and induration at the application site, while systemic adverse events corresponded to fewer, shivering, fatigue, headache, myalgia and arthralgia [25].

All participants signed a voluntary informed consent form, providing demographic and clinical data and participation consent.

### 2.6. Statistical Analysis

Results were presented as frequency (percent), median (range) and mean ± standard deviation (SD). Changes in the anti-spike IgG during the observed period were examined with a linear mixed effects modeling approach using R package lme4 version 1.1–26. The models fit was assess using Akaike’s information criterion (AIC). Results were graphically presented using the R package ggplot2 version 3.3.2. Logistic regression was used as the method for analyzing binary outcomes (local and systemic AE) and potential predictors. Independent variables that were significant (*p* < 0.1) in univariate logistic regression (ULRA) models were used as the independent variables in the multivariate logistic regression (MLRA) model. Hosmer and Lemeshow goodness-of-fit test was performed to determine how well the model fits the data. Multicollinearity was checked with Variance Inflation Factors (VIF). All *p*-values less than 0.05 were considered significant. Statistical data analysis was performed using IBM SPSS Statistics 22 (IBM Corporation, Armonk, NY, USA) and R-4.0.0 software (The R Foundation for Statistical Computing, Vienna, Austria).

### 2.7. Ethical Statement

The Ethics Committee of the Institute of Public Health of Serbia “Dr Milan Jovanovic Batut” approved the study (No 5139/1). Personal identifiers of study participants were coded, and patient records were anonymized and de-identified prior to analysis to maintain the confidentiality.

## 3. Results

### 3.1. Characteristics of the Study Participants

Out of 300 study participants, 185 (61.7%) were female. The mean age for all participants was 52.7 (SD = 14.3). Pfizer-BioNTech was the most common COVID-19 vaccine used as a booster dose by 226 (75.3) participants, while 60 (20%) and 14 (4.7%) used Sinopharm BBIBP-CorV and Sputnik V, respectively. The homologous booster immunization was used in 127 (42.3%) participants, while heterologous in 173 (57.7%). Other characteristics are described in Table 1.

The most common vaccine combinations (primary vaccination series and booster doses) used are presented in Table 2.

Almost three-fourths of the participants, i.e., 214 (71.3%), reported local adverse events 7 days after the booster dose, while 100 (33.3%) of them reported systemic adverse events. Adverse events after the first dose of the COVID-19 vaccine were reported by 50 participants (16.7%) and by 51 (17.0%) participants after the second dose of the COVID-19 vaccine.

### 3.2. Analysis of Immunogenicity

**Figure 1:**
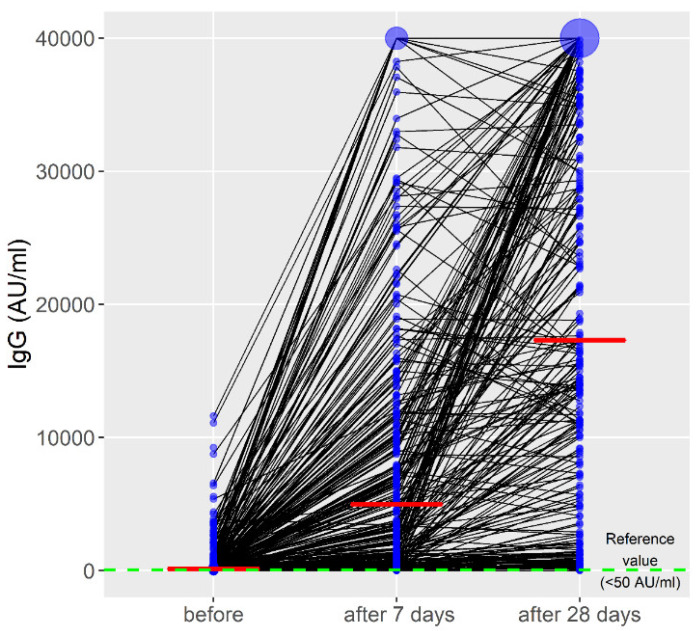
Titers of anti-spike immunoglobulin G during the observed period. The red line represents the median IgG values; the blue circle represents the number of cases with the same IgG values.

The initial antibody titer after 6 months of the primary vaccination by all vaccines was 134.0 (range, 0–11615.8). During the study period, there was a significant increase in anti-spike Ig G (B = 9757.4; *p* < 0.001, *η*^2^ = 0.53) (Figure 1).

**Figure 2:**
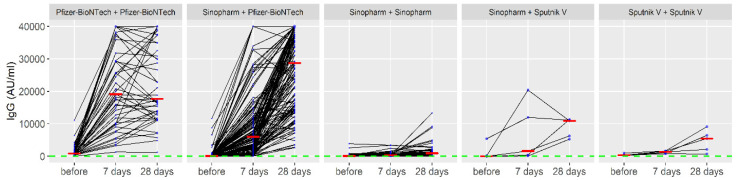
Titers of anti-spike immunoglobulin G in applied vaccine combinations (primary vaccination series and booster dose) during the observed period. The red line represents the median IgG values; the blue circle represents the number of cases with the same IgG values.

When applying the booster dose within the homologous regimen, the average increase in the anti-S-IgG was 8782.2 (after 7 days), while after 28 days it was 1213.9. Within the heterologous regimen, the average increase in the anti-S-IgG was 9179.5 (after 7 days), while after 28 days it was 16,728.1. Titers of anti-S-IgG in all applied vaccine combinations (primary vaccination series and booster dose) during the observed period are presented in Figure 2.

Based on the results presented in Figure 2, the Pfizer-BioNTech—Pfizer-BioNTech combination (primary vaccination series and booster dose) showed the biggest effect. In addition, the administration of the third dose of Pfizer-BioNTech in those who had previously received two doses of the Sinopharm vaccine as a primary series has also shown a substantial booster effect. On the other hand, other regimens (e.g., Sputnik–Sputnik) did not induce a significant response, although a modest rise in antibody levels has been observed in some individuals. Interestingly, a decrease in IgG values 28 days after the booster dose compared to 7 days after the booster dose of COVID-19 vaccines was observed in 17% (44/259) of participants who received the homologous booster of Pfizer-BioNTech. This decrease was associated with confirmed COVID-19 infections (OR = 5.64; 95%CI = 1.16–19.67), homologous vaccines (OR = 4.89; 95%CI = 2.21–10.82) and adverse events after the second dose (OR = 2.53; 95%CI = 1.03–5.84). An analysis of the decrease in IgG values 28 days after the booster dose compared to 7 days after the booster dose of COVID-19 vaccines is presented in Table 3.

Final multivariable model with IgG values as a dependent variable had marginal *R*^2^ = 0.52 and conditional *R*^2^ = 0.55. Sinopharm BBIBP-CorV booster dose (B = −10,891.2) and Sputnik V booster dose (B = −9001.6) in relation to the Pfizer-BioNTech booster dose as a reference category, 65 years and over (B = −3449.1) and currently smoking (B = −2577.0) were found to be independently associated with a lower level of anti-S-IgG, while adverse events after the second dose of the COVID-19 vaccine (B = 3093.3) were found to be independently associated with a higher level of anti-S-IgG (Table 4).

### 3.3. Analysis of Adverse Events after Booster Dose

All recorded adverse events were detected 7 days after the booster dose, while there were no local and systemic adverse events 28 days after the booster dose.

Local adverse events 7 days after the booster dose were reported in 214 (74.3%) of the study participants. The most common events were pain at the application site in 211 (70.3%), induration in 59 (19.6%), swelling in 55 (18.3%) and redness in 42 (14.0%).

Female sex (OR = 2.79; 95%CI = 1.37–5.72) and homologous booster COVID-19 vaccinations (OR = 4.84; 95%CI = 1.98–11.78) were independently associated with more intense local adverse events 7 days after the booster dose, while older age, i.e., 65+ years (OR = 0.31; 95%CI = 0.11–0.87) and Sinopharm BBIBP-CorV booster dose (OR = 0.23; 95%CI = 0.07–0.67) in relation to the Pfizer-BioNTech booster dose as a reference category were found to be independently associated with less intense local adverse events 7 days after the booster dose (Table 5).

### 3.4. Systemic Adverse Events 7 Days after the Booster Dose

Systemic adverse events 7 days after the booster dose were reported in 100 (34.7%) participants. The most common events were fatigue experienced by 53 (17.7%), headaches in 30 (10.0%), myalgia by 29 (9.6%), shivering by 25 (8.3%), arthralgia by 25 (8.3%) and fever by 23 (7.6%).

Female sex (OR = 1.77; 95%CI = 1.01–3.12), previous COVID-19 infection (OR = 3.62; 95%CI = 1.13–11.63) and adverse events after the second dose of the COVID-19 vaccine (OR = 2.66; 95%CI = 1.33–5.32) were found to be independently associated with more intense systemic adverse events 7 days after the booster dose (Table 6).

## 4. Discussion

The results of our study indicate that a heterologous vaccine regimen led to a significantly stronger booster of the humoral immune response 28 days after vaccine administration than the homologous one. Pfizer-BioNTech vaccine, as a booster, induced the strongest humoral immune response. Other predictors of a stronger humoral immune response were younger age, non-smoking status and the occurrence of adverse events after the second dose of the COVID-19 vaccine within the primary series of COVID-19 immunization. The diagnosis of malignant disease was a predictor of a weaker humoral immune response, while the presence of other chronic diseases was not found as a significant predictor. Receiving a booster dose of Pfizer-BioNTech, Sputnik V and Sinopharm BBIBP-CorV vaccines turned out to be safe and well tolerated. A low percentage of adverse events were reported up to the seventh day after receiving the booster dose of any of the given vaccines, while no adverse events were reported between the seventh and 28th day after the booster dose. Regardless of the type of adverse event (local or systemic), all reactions were mild to moderate in severity, without serious adverse events reported.

Other studies comparing homologous and heterologous vaccine regimens showed that heterologous vaccine administration boosted the humoral immune response significantly and strongly [15,16,17,18,26,27,28,29,30,31]. In line with our results, the advantage of a heterologous vaccine regimen with stronger humoral response is evident and can be explained by the administration of an mRNA-based vaccine as a second or booster dose [15,16,17,18,26,27,28,29,30,31]. Pfizer-BioNTech vaccine was shown to induce stronger humoral immune response (production of anti-receptor-binding domain (RBD) IgG, anti-spike protein IgG, IgA antibodies) in healthy individuals, as well as a two-fold higher T cell response [28]. Study by Petrovic et al. also demonstrated that the Pfizer-BioNTech vaccine induced higher antibody levels against S protein 28 days after vaccination with two doses, compared to Sputnik V and Sinopharm BBIBP-CorV [32]. Although less potent compared to other two vaccines, immune response after Sinopharm BBIBP-CorV was similar to the response measured in convalescents. Similarly, in a study from Mongolia, the strongest anti-spike antibody ACE2 blocking activity was noticed in the Pfizer-BioNTech vaccine, followed by AstraZeneca and Sputnik V vaccines, while the lowest levels were detected after the administration of Sinopharm BBIBP-CorV vaccine [26]. Taking all this into account, it is not surprising that a heterologous booster with Pfizer/BioNTech vaccine in our study induced stronger humoral response in subjects who received Sinopharm BBIBP-CorV vaccine as a primary series in comparison with the homologous booster regimen. In concordance with our findings, heterologous booster vaccination with the Pfizer/BioNTech vaccine in COVID-19-naïve individuals who had received two doses of the Sinopharm BBIBP-CorV vaccine was not only found to be safe and well tolerated, but it was also significantly associated with higher anti-spike IgG geometric mean titers compared to that after homologous Pfizer/BioNTech immunization in COVID-19-naïve individuals [15]. Likewise, heterologous booster with Pfizer/BioNTech vaccine after Sinovac CoronaVac, another inactivated vaccine, boosted anti-spike IgG median titers by a factor of 46.6, compared to only 1.7 times increase after the homologous booster, although this was not observed for the humoral response against nucleocapsid (N) protein of SARS-CoV-2 [33].

Our study results as well as previous studies indicate that the Sinopharm BBIBP-CorV vaccine led to a slower and lower increase in anti-spike IgG 28 days after booster dose administration [15,16,17,18,23]. We examined the increase in titer of anti-spike IgG (anti-S-IgG), without testing the presence of antibodies to the SARS-CoV-2 N antigen produced only after receiving the Sinopharm BBIBP-CorV vaccine. A study by Dashdorj et al. found that individuals with higher anti-N antibodies, in addition to anti-spike antibody levels, have stronger ACE2-blocking antibody activity [26] in relation to those without anti-N antibodies. As the Sinopharm BBIBP-CorV vaccine induced T cell response targeting N and membrane (M) proteins similar to cell-mediated immune response in patients who recovered from COVID-19 [25], not the case with Pfizer-BioNTech vaccine, isolated the measurement of S protein-binding antibody titers after the COVID-19 vaccines is not sufficient to conclude the immune efficacy of Sinopharm BBIBP-CorV and other inactivated vaccines [28].

However, a correlation has been observed between higher antibody titers induced by different types of COVID-19 vaccines and a higher degree of protection against all known SARS-CoV-2 variants, despite various uncontrolled variables across the studies [29,30]. According to the latest results from the meta-analysis, the efficacy of vaccines against symptomatic infection caused by different variants of SARS-CoV-2 is reduced to below 50% within the first year after vaccination for some vaccines [34]. The initial levels of binding antibodies after two doses of inactivated vaccines begins to decline after 90 and 180 days, and after six months they are near or below the limit of seropositivity [30], which is similar to our results. It has been shown that a booster immunization enables greater neutralization of SARS-CoV-2 variants compared to primary vaccination [31]. The study by Zeng et al. demonstrated that administration of the third dose of inactivated vaccine after a longer time interval from the second dose (8 vs. 2 months) led to a stronger increase in the titer of neutralizing antibodies, which is approximately three to five times higher than titers observed 28 days after the second dose, while the reactogenicity of the third dose did not differ from the reactogenicity of the previous two doses [30]. In the present study, a booster dose was administered to participants after a minimum of 6 months, which could explain the strong response, especially after heterologous regimen. Regarding various factors that might affect immune response to vaccines, the association of older age, smoking and malignancy with lower humoral immune responses following the COVID-19 vaccine observed in our study is probably linked with different levels of immune suppression in those individuals, and such associations were also observed in previous studies [30,31,32]. Interestingly, we have observed a decline in IgG levels in the period between day 7 and day 28 post-vaccination in some participants in our study, mostly those who received Pfizer/BioNTech in homologous regimen. Although we are not able to explain this observation, it is worth mentioning that, in addition to the administration of homologous vaccines, the decrease in IgG values was also associated with confirmed COVID-19 infections and adverse events after the second dose.

Concerning the safety, the results of our study indicate that the frequency and severity of adverse events after receiving a booster dose of COVID-19 vaccine is neither higher nor different from the adverse events that occurred during the primary vaccination. In our cohort, female gender and the homologous booster dose of the COVID-19 vaccine were predictors of more intense local adverse events, and previous COVID-19 infection and adverse events after the second dose of the COVID-19 vaccine were predictors of more intense systemic adverse events notified within 7 days. Older age and the Sinopharm BBIBP-CorV vaccine used as a third dose were predictors of less intense local adverse events within 7 days. Our results are consistent with the previous studies which examined the frequency of adverse reactions to combinations of different vaccine types. These studies have shown that the adverse reactions are more common after the Pfizer-BioNTech and AstraZeneca vaccine compared to inactivated vaccines and are generally mild to moderate in severity [15,18,34]. It has also been noticed that the frequency of adverse events after the vaccine against COVID-19 is higher among convalescents [34,35]. Additionally, the occurrence of side effects is more common in those who experienced side effects when receiving the previous dose of the COVID-19 vaccine [34,35].

In our study, adverse events observed after receiving any of the three COVID-19 vaccines (Pfizer/BioNTech, Sputnik V and Sinopharm BBIBP-CorV) were mild to moderate. In general, the incidence and severity of adverse events after inactivated vaccines are the lowest compared to those that follow the administration of mRNA or vector vaccines [34,35]. Pain at the site of vaccine injection and fatigue were the most reported local and systemic adverse events for all COVID-19 vaccines [17,18,31,34]. The fever is more often reported after receiving the Sputnik V vaccine [35]. In our study, it was noticed that the reactogenicity of the third dose did not differ significantly from the reactogenicity after the previous two doses, regardless of the type of vaccine applied, in accordance with the data from the literature [17,18,22,31].

Our study is the first one in Serbia which evaluated the immunogenicity and safety of administration of the booster dose and is one of the rare studies that assessed heterologous regimens that included the Pfizer/BioNTech, Sputnik V and Sinopharm BBIBP-CorV vaccines. However, this study has several limitations. It included a limited number of respondents due to the funding restraints. Therefore, we did not manage to recruit enough subjects to have equal distribution between the groups. Moreover, limited quantities of the Sputnik V vaccine were available for booster administration during the study implementation resulting in a small number of subjects receiving that vaccine as a booster, despite the interest among the participants. As a result, we cannot draw any firm conclusions about the effects of the Sputnik V vaccines used as a booster. Thus, a larger sample size is needed to explore these, and other factors associated with immune response and adverse events following immunization. Despite its limitations, our study provided baseline data about the safety and immunogenicity of mix and match strategies of the COVID-19 booster immunization in Serbia.

## 5. Conclusions

The application of the booster dose significantly increased antibody titers against spike protein, especially 28 days after the applied heterologous regimen. The observed increased immunogenicity of the booster dose was not accompanied by an increase in reactivity. Our study results also indicated the presence of low antibody titers recorded six months after the second dose of four COVID-19 vaccines administered.

## Figures and Tables

**Table 1:** Characteristics of the study participants (*n* = 300).

Characteristics	N	%
Sex		
Male	115	38.3
Female	185	61.7
Age category		
18–44	85	28.3
45–64	143	47.7
65+	72	24.0
Education		
High school or lower	65	21.6
University or higher	235	78.3
Employment		
Employed	221	73.7
Retired	63	21.0
Student/Unemployed	16	5.3
Marital status		
Married/cohabitation	194	64.7
Single	106	35.3
Residence		
Urban/Suburb	280	93.3
Rural	20	6.7
Currently smoking	103	34.3
Alcohol use	70	23.4
Concomitant diseases		
Cardiac *	109	36.3
COPD ** and asthma	22	7.3
Diabetes ***	40	13.3
Thyroid	35	11.7
Malignant ****	15	5.0
Previous COVID-19 infection	19	6.3
Immunosuppressive treatment	4	1.3
Allergy to food and drugs	53	17.7
Type of booster dose of COVID-19 vaccine		
Sinopharm BBIBP-CorV	60	20.0
Pfizer-BioNTech	226	75.3
Sputnjik V	14	4.7
Homologous booster vaccination	127	42.3
IgG baseline titer	300	100.0
Adverse events after first dose of COVID-19 vaccine	50	16.7
Adverse events after second dose of COVID-19 vaccine	51	17.0
Local adverse events 7 days after booster dose	214	74.3
Systemic adverse events 7 days after booster dose	100	34.7

* Arterial hypertension, ischemic heart disease and cardiac insufficiency; ** chronic obstructive pulmonary disease; *** all types of diabetes; **** all localizations.

**Table 2:** Distribution of applied vaccine combinations (primary vaccination series and booster doses, *n* = 300).

Primary Vaccination Series and Booster Doses	N	%
Sinopharm BBIBP-CorV + Sinopharm BBIBP-CorV	59	19.7
Sinopharm BBIBP-CorV + Pfizer-BioNTech	163	54.3
Sinopharm BBIBP-CorV + Sputnik V	8	2.7
Pfizer-BioNTech + Pfizer-BioNTech	62	20.7
Pfizer-BioNTech + Sinopharm BBIBP-CorV	1	0.3
Sputnik V + Sputnik V	6	2.0
Sputnik V + Pfizer-BioNTech	1	0.3

**Table 3:** Univariate and multivariate analysis of the decrease in IgG values 28 days after the booster dose compared to 7 days after the booster dose of COVID-19 vaccines (*n* = 259).

Characteristics	Univariate		Multivariate	
	OR (95% CI)	*p*-Value	OR (95% CI)	*p*-Value
Sex (f vs. m)	1.46 (0.72–2.96)	0.292		
Age category	0.96 (0.94–0.98)	0.001		
18–44	ref		ref	
45–64	0.75 (0.37–1.52)	0.423	0.91 (0.40–2.04)	0.810
65+	0.10 (0.02–0.43)	0.002	0.20 (0.04–1.01)	0.051
Education (university vs. high)	1.93 (0.77–4.83)	0.161		
Employment				
Employed	ref			
Retired	0.12 (0.03–0.53)	0.005		
Student/Unemployed	-	-		
Marital status (single vs. married)	0.89 (0.45–1.8)	0.754		
Residence (urban vs. rural)	0.93 (0.26–3.4)	0.917		
Currently smoking	1.26 (0.64–2.48)	0.497		
Alcohol use	0.97 (0.48–1.96)	0.935		
Concomitant diseases				
Cardiac *	0.27 (0.12–0.64)	0.003	0.38 (0.14–1.01)	0.053
COPD ** and asthma	0.23 (0.03–1.77)	0.158		
Diabetes ***	0.57 (0.13–2.54)	0.457		
Thyroid	0.95 (0.34–2.63)	0.924		
Malignant ****	0.44 (0.06–3.5)	0.437		
Previous COVID-19 infection	5.75 (1.9–17.37)	0.002	5.64 (1.16–19.67)	0.007
Immunosuppressive treatment	1.67 (0.17–16.49)	0.659		
Allergy to food and drugs	1.85 (0.87–3.94)	0.110		
Type of booster dose of COVID-19 vaccine				
Pfizer-BioNTech	ref			
Sinopharm BBIBP-CorV	0.59 (0.23–1.48)	0.256		
Sputnik V	2.00 (0.49–8.15)			
Homologous booster COVID-19 vaccinations	0.21 (0.1–0.43)	<0.001	4.89 (2.21–10.82)	<0.001
Adverse events after first dose of COVID-19 vaccine	3.21 (1.51–6.8)	0.002		
Adverse events after second dose of COVID-19 vaccine	3.34 (1.57–7.11)	0.002	2.53 (1.03–5.84)	0.043

* Arterial hypertension, ischemic heart disease and cardiac insufficiency; ** chronic obstructive pulmonary disease; *** all types of diabetes; **** all localizations.

**Table 4:** Univariate and multivariable linear mixed-effect regression models with IgG values as a dependent variable.

Characteristics	Univariate		Multivariate	
	B	*p*-Value	B	*p*-Value
Sex (f vs. m)	570.7	0.538		
Age category	−152.8	<0.001		
18–44	ref		ref	
45–64	−1646.2	0.118	−1583.0	0.057
65+	−4381.7	<0.001	−3449.1	0.001
Education (university vs. high)	1806.1	0.097	−878.9	0.297
Employment				
Employed	ref			
Retired	−3717.6	0.001		
Student/Unemployed	1686.6	0.394		
Marital status (single vs. married)	1435.9	0.126		
Residence (urban vs. rural)	1424.9	0.431		
Currently smoking	−1800.7	0.057	−2577.8	<0.001
Alcohol use	1953.8	0.034	1221.0	0.084
Concomitant diseases				
Cardiac *	−2265.6	0.015	377.0	0.642
COPD ** and asthma	−2506.5	0.141		
Diabetes ***	−4100.0	0.015	−1787.9	0.177
Thyroid	−308.2	0.831		
Malignant ****	−3872.8	0.070	−3378.0	0.039
Previous COVID-19 infection	1841.0	0.333		
Immunosuppressive treatment	−8626.3	0.025	−1895.2	0.512
Allergy to food and drugs	−126.1	0.915		
Type of booster dose of COVID-19 vaccine				
Pfizer-BioNTech	ref		ref	
Sinopharm BBIBP-CorV	−11,696.8	<0.001	−10,891.2	<0.001
Sputnik V	−8156.4	<0.001	−9001.6	<0.001
Homologous booster COVID-19 vaccinations	4338.8	<0.001	−1026.7	0.244
Adverse events after first dose of COVID-19 vaccine	628.9	0.603		
Adverse events after second dose of COVID-19 vaccine	4472.8	<0.001	3093.3	0.001

* Arterial hypertension, ischemic heart disease and cardiac insufficiency; ** chronic obstructive pulmonary disease; *** all types of diabetes; **** all localizations.

**Table 5:** Univariate and multivariate analysis of local adverse events 7 days after the booster dose of COVID-19 vaccines.

Characteristics	Univariate		Multivariate	
	OR (95% CI)	*p*-Value	OR (95% CI)	*p*-Value
Sex (f vs. m)	1.89 (1.11–3.23)	0.020	2.79 (1.37–5.72)	0.005
Age category				
18–44	ref		ref	
45–64	0.72 (0.36–1.46)	0.362	0.80 (0.33–1.94)	0.618
65+	0.28 (0.13–0.60	0.001	0.31 (0.11–0.87)	0.026
Education (university vs. high)	1.67 (0.91–3.08)	0.098	1.21 (0.56–2.60)	0.625
Employment				
Employed	ref			
Retired	0.82 (0.25–2.66)	0.739		
Student/Unemployed	0.40 (0.22–0.74)	0.003		
Marital status (single vs. married)	1.71 (0.96–3.06)	0.071	1.14 (0.55–2.35)	0.724
Residence (urban vs. rural)	1.04 (0.36–2.98)	0.949		
Currently smoking	1.06 (0.61–1.85)	0.844		
Alcohol use	1.00 (0.57–1.78)	0.988		
Concomitant diseases				
Cardiac *	0.37 (0.22–0.64)	<0.001	0.49 (0.24–1.02)	0.056
COPD ** and asthma	0.72 (0.28–1.85)	0.496		
Diabetes ***	0.62 (0.26–1.53)	0.302		
Thyroid	1.29 (0.50–2.94)	0.675		
Malignant ****	0.38 (0.13–1.18)	0.095	0.37(0.10–1.40)	0.144
Previous COVID-19 infection	2.52 (0.56–11.36)	0.229		
Immunosuppressive treatment	0.34 (0.05–2.46)	0.285		
Allergy to food and drugs	1.01 (0.51–2.03)	0.971		
Type of booster dose of COVID-19 vaccine				
Pfizer-BioNTech	ref		ref	
Sinopharm BBIBP-CorV	0.11 (0.06–0.22)	<0.001	0.23 (0.08–0.67)	0.008
Sputnik V	0.37 (0.11–1.30	0.120	0.56 (0.12–2.61)	0.461
Homologous booster COVID-19 vaccinations	6.68 (3.65–12.22)	<0.001	4.84 (1.98–11.78)	0.001
Adverse events after first dose of COVID-19 vaccine	1.20 (0.58–2.49)	0.630		
Adverse events after second dose of COVID-19 vaccine	1.66 (0.76–3.61)	0.201		
IgG baseline titer	1.0001 (0.9999–1.0003)	0.370		
IgG titer 7 days after booster dose	1.00004 (1.00001–1.00008)	0.012	0.99999 (0.99995–1.00002)	0.495

* Arterial hypertension, ischemic heart disease and cardiac insufficiency; ** chronic obstructive pulmonary disease; *** all types of diabetes; **** all localizations.

**Table 6:** Univariate and multivariate analysis of systemic adverse events 7 days after the booster dose of COVID-19 vaccines.

Characteristics	Univariate		Multivariate	
	OR (95% CI)	*p*-Value	OR (95% CI)	*p*-Value
Sex (f vs. m)	1.76 (1.05–2.96)	0.031	1.77 (1.01–3.12)	0.046
Age category				
18–44	ref			
45–64	0.66 (0.37–1.16)	0.152		
65+	0.67 (0.35–1.31)	0.248		
Education (university vs. high)	1.15 (0.63–2.09)	0.646		
Employment				
Employed	ref			
Retired	0.83 (0.45–1.53)	0.547		
Student/Unemployed	1.88 (0.68–5.21)	0.227		
Marital status (single vs. married)	1.16 (0.70–1.92)	0.564		
Residence (urban vs. rural)	3.01 (0.85–10.58)	0.086	3.10 (0.83–11.56)	0.092
Currently smoking	0.72 (0.43–1.21)	0.220		
Alcohol use	1.00 (0.59–1.69)	0.998		
Concomitant diseases				
Cardiac *	0.85 (0.51–1.41	0.527		
COPD ** and asthma	1.33 (0.55–3.23)	0.527		
Diabetes ***	1.50 (0.63–3.54)	0.360		
Thyroid	1.41 (0.66–3.01)	0.374		
Malignant ****	0.56 (0.15–2.05)	0.373		
Previous COVID-19 infection	3.37 (1.19–9.57)	0.022	3.62 (1.13–11.63)	0.031
Immunosuppressive treatment	0.34 (0.05–2.46)	0.285		
Allergy to food and drugs	1.40 (0.75–2.60)	0.287		
Type of booster dose of COVID-19 vaccine				
Pfizer-BioNTech	ref		ref	
Sinopharm BBIBP-CorV	0.38 (0.18–0.78)	0.008	0.59 (0.26–1.31)	0.194
Sputnik V	1.63 (0.51–5.21)	0.413	2.34 (0.66–8.25)	0.187
Homologous booster COVID-19 vaccinations	1.29 (0.79–2.12)	0.311		
Adverse events after first dose of COVID-19 vaccine	1.59 (0.84–2.98)	0.152		
Adverse events after second dose of COVID-19 vaccine	3.43 (1.82–6.47)	<0.001	2.66 (1.33–5.32)	0.006
IgG baseline titer	1.00006 (0.99990–1.00023)	0.460		
IgG titer 7 days after booster dose	1.00004 (1.00002–1.00006)	<0.001	1.00002 (0.99999–1.00005)	0.064

* Arterial hypertension, ischemic heart disease and cardiac insufficiency; ** chronic obstructive pulmonary disease; *** all types of diabetes; **** all localizations.

## Data Availability

The supporting data for the study findings are available from the corresponding author upon request.
